# Quercetagetin alleviates inflammatory osteoclastogenesis and collagen antibody-induced arthritis via Nrf2 signaling and Pten/AKT/Nfatc1 axis

**DOI:** 10.1186/s13075-025-03522-x

**Published:** 2025-03-08

**Authors:** Haojue Wang, Tao Yuan, Jingpeng Wang, Dengju Li, Wayne Yuk-wai Lee, Ziqing Li, Shui Sun

**Affiliations:** 1 Department of Joint Surgery, Cheeloo College of Medicine, Shandong Provincial Hospital, Shandong University, Jinan, Shandong 250012 China; 2 Department of Joint Surgery, Shandong Provincial Hospital Affiliated to Shandong First Medical University, Jinan, Shandong 250021 China; 3 Heze Sports School, Heze, Shandong 274000 China; 4 Orthopaedic Research Laboratory, Medical Science and Technology Innovation Center, Shandong First Medical University & Shandong Academy of Medical Sciences, Jinan, Shandong 250117 China; 5 Department of Orthopaedics and Traumatology, The Chinese University of Hong Kong, Hong Kong, China; 6 SH Ho Scoliosis Research Laboratory, Joint Scoliosis Research Centre of the Chinese University of Hong Kong and Nanjing University, The Chinese University of Hong Kong, Hong Kong, China; 7 Li Ka Shing Institute of Health Sciences, The Chinese University of Hong Kong, Hong Kong, China

**Keywords:** Quercetagetin, Flos eriocauli, Lipopolysaccharide, Rheumatoid arthritis, Osteoclast

## Abstract

**Purpose:**

Quercetagetin, a flavonoid derived from the natural herb Flos eriocauli, is used in traditional Chinese medicine for its fire-purging (anti-inflammation) and wind-expelling (pain-alleviating) properties. However, its potential effects concerning rheumatoid arthritis (RA) remain underexplored. This study was designed to elucidate the potential associations between Quercetagetin and RA, establishing the therapeutic potential of Quercetagetin and related mechanisms in RA treatment.

**Methods:**

Network pharmacology was conducted to decipher related targets and signaling pathways between Quercetagetin and RA. In vitro assays were then conducted to explore the effects of Quercetagetin on osteoclast cell behaviors and corresponding signaling pathways. In vivo study further validated the therapeutic effect of Quercetagetin in collagen antibody-induced arthritis (CAIA) mice.

**Results:**

The network pharmacological analysis indicated an intimate correlation of Quercetagetin with RA-related inflammatory osteolysis treatment. Pertaining to biological validations, 2 µM of Quercetagetin successfully inhibited LPS-driven osteoclast differentiation and function. qPCR assay and Western blot analyses denoted parallel changes in osteoclastic marker genes and proteins. Further mechanism study uncovered the effect of Quercetagetin in stimulating the Nrf2/Keap1 signaling pathway and moderating the Pten/AKT/Nfatc1 axis in osteoclasts. In vivo study revealed 40 mg/kg Quercetagetin every day could significantly relief joint destruction in CAIA mice.

**Conclusions:**

Our study presents Quercetagetin ‘s therapeutic potential in treating RA, outlining its effects and potential mechanisms in suppressing LPS-induced osteoclast activity, and alleviating inflammatory bone destruction in CAIA model, thereby laying the groundwork for further translational research on Quercetagetin and Flos eriocauli in RA treatment.

**Supplementary Information:**

The online version contains supplementary material available at 10.1186/s13075-025-03522-x.

## Highlights

Quercetagetin extracted from Flos eriocauli displayed brilliant anti-osteoclast and anti-inflammation potential during rheumatoid arthritis.

Quercetagetin significantly repressed lipopolysaccharide-stimulated osteoclast behavior from phenotypical and molecular level.

Quercetagetin mitigated inflammatory bone destruction in collagen antibody-induced arthritis.

**Supplementary Information:**

The online version contains supplementary material available at 10.1186/s13075-025-03522-x.

## Introduction

Rheumatoid arthritis (RA), a widespread, prolonged, and systematic autoimmune disease, is characterized by irrevocable impairment entailing multiple small joints [1]. While the pathophysiology of RA is still somewhat elusive, plentiful relevant contributors have been raised, including hormonal effects, female sex, smoking, and genetic susceptibilities [1, 2]. Currently, the incidence of RA is continuously escalating which involves about 20 million people globally, accompanying with an experience of 50% greater risk of cardiovascular mortality in the RA population [3]. Great progress has been reached in early RA diagnostics and etiological studies, providing the opportunity for personalized and symptom-modified interventions for RA patients [4, 5]. Despite being coexistent with multiple extra-articular symptoms, such as pulmonary, cardiovascular, gastrointestinal, renal, and neurological comorbidities [3], the most eminent and concerning characteristic of RA remains the articular pathological alterations [6, 7]. Presently, western medicine adopts non-steroid anti-inflammation drugs (NSAIDs), disease-modifying antirheumatic drugs (DMARDs), and corticosteroids as primary therapies for RA. However, due to the limited targeting capabilities and significant side effects accompanied with these approaches, there is a pressing need to explore novel therapeutics grounded in a deeper understanding of pathological mechanisms, aiming to delay or attenuate irreversible joint degeneration and alleviate relevant clinical symptoms [8].

The intensified inflammatory osteolysis and para-articular osteoporosis mediated by hyperactivated osteoclasts under RA conditions, greatly contribute to the articular anomalies and disabilities in RA patients. As a central meditator in inflammatory disorders, Reactive Oxygen Species (ROS) is a recognized second messenger of osteoclastogenesis, propelling downstream signaling transduction of the Receptor Activator of Nuclear Factor Kappa-B Ligand (RANKL)-mediated signaling pathway [9]. Ample evidence has raised ROS an important second messenger in activating PI3K/AKT, MAPK and NF-κB and calcium signaling pathway in osteoclast differentiation [9]. Among the agents recognized for mimicking the inflammatory condition of RA, Lipopolysaccharide (LPS), a major component of the gram-negative bacteria wall, has proven to stimulate osteoclast cellular activities through escalating ROS levels and promoting various inflammatory factors [10–12]. Although osteoclast-inhibitory biological agents, including cytokines blockers corresponding to LPS-stimulated inflammatory factors [3], have exhibited superior targeting ability and therapeutic efficiency in RA treatment, these treatments also encountered challenges, for instance, a ceiling effect is verified, with only 10–20% of RA patients experiencing symptom remissions. Furthermore, prolonged use may lead to severe infections, and elderly patients often exhibit reduced efficacy and safety [13–16].During the decades, traditional Chinese medicine (TCM) and its derivatives have demonstrated efficacy in both clinical practice and experimental verification of RA [17]. For example, the Simiao pill, Soufeng Sanjie formula, and Guizhi Shaoyao Zhimu decoction can all alleviate inflammation, bone erosion, cartilage degeneration, synovial hyperplasia, and immune imbalance in RA [10]. Bioactive compounds extracted from natural herbs used in the formula have been demonstrated to possess genuine therapeutic functions, including anti-inflammatory, antioxidant, antidiabetic, and antibacterial [18, 19]. Considering that chronic inflammatory circumstances and oxidative stress are common characteristics of RA, bioactive compounds possess well anti-inflammatory and antioxidant properties may manifest corresponding therapeutic effects in RA treatment.

Flos Eriocauli, serving as the fire purging and wind expelling agent, has demonstrated anti-inflammation and ROS clearance potential [20]. A study revealed abundant flavonoid compounds contained in Flos Eriocauli [21]. For RA patients, flavonoid compounds have demonstrated well anti-osteoclastogenic, antioxidant, and anti-inflammatory effects [22, 23]. Therefore, although the practical effect and molecular mechanisms were rarely reported in prior studies, Flos Eriocauli and its derivatives hold great promise in RA treatment. Owing to significant progress in big data and bioinformatic methodologies over the past decade, network pharmacology now enables the systematic and comprehensive analysis of multi-drug, multi-pathway, and multi-disease frameworks concurrently [24, 25]. By employing methods such as targets prediction from multiple databases, network construction of protein-protein interaction (PPI), enrichment analyses based on Gene Ontology (GO) and Kyoto Encyclopedia of Genes Genomes (KEGG), and molecular docking, network pharmacology serves as a reliable instrument for analyzing properties of bioactive compounds from TCM as well as exploring drug targets and their associated molecular mechanisms. Consequently, network pharmacology could be a useful tool for revealing the potential efficacy of Flos Eriocauli and its major derivatives in RA treatment.

In this study, by utilizing network pharmacology, we aimed to conduct an analysis on Quercetagetin, a major bioactive flavonoid extraction of Flos Eriocauli, and endeavored to decipher the potential association between Quercetagetin and RA, predetermining the conceivable therapeutic targets and signaling pathways. Subsequent biological validations were performed based on the results from network pharmacology, attempting to explore the effect of Quercetagetin on LPS-induced osteoclast differentiation and function, and validate its effect in collagen antibody-induced arthritis (CAIA) model, thus to establish Quercetagetin as a potential candidate for RA treatment through acting on corresponding signaling pathways.

## Methods and materials

### Network Pharmacological analysis

#### Bioactive compounds in Flos eriocauli

All components of Flos Eriocauli were obtained from the Traditional Chinese Medicine Systems Pharmacology Database and Analysis Platform (TCMSP) (http://lsp.nwu.edu.cn/tcmsp.php) [26]. The bioactive compounds were then screened according to parameters including oral bioavailability (OB), drug-likeness (DL), and blood-brain barrier (BBB).

#### Potential targets of Quercetagetin and RA

The potential targets of Quercetagetin were extracted from databases including HERB (http://herb.ac.cn/), TCMSP, and SwissTarget (http://swisstargetprediction.ch/). The disease targets related to RA were obtained from the Comparative Toxicogenomics Database (CTD) (http://ctdbase.org/). Overlapping targets (shared gene) of Quercetagetin and RA were then screened out with the Uniport database (https://www.UniProt.org/) [27] using the term “Homo sapiens” as a filter.

#### Protein-protein interaction (PPI) network and enrichment analysis

To construct the PPI network, gene targets were analyzed with STRING online database (https://string-db.org/) [28]. By defining organisms as “Homo sapiens” and confidence score as 0.4 (medium confidence), the results of the PPI analysis were then downloaded or re-visualized with the Cytoscape software (version 3.10.0) [29]. Gene Ontology (GO) and Kyoto Encyclopedia of Genes and Genomes (KEGG) analyses were then performed using the Database for Annotation, Visualization, and Integrated Discovery (DAVID) (https://david.ncifcrf.gov/home.jsp) [30], with the limitation of species to “Homo sapiens”. Terms with *P* < 0.05 were included and subjected for further analysis. The Bioinformatics platform (https://www.bioinformatics.com.cn/) was subsequently used to facilitate the visualization of the GO biological process, KEGG pathways, and interrelationship among targets and terms.

#### Molecular Docking

The molecular structure of Quercetagetin was obtained from PubChem (https://pubchem.ncbi.nlm.nih.gov/) [31]. The three-dimensional structures of the target proteins including AKT1 (3QKM), NFKB1 (2O61), PTGS2 (5F19), and TNF (6 × 18) were acquired from the Protein Data Bank (PDB) database (https://www.rcsb.org/) [32]. Molecular docking between Quercetagetin with these protein targets was performed employing the MOE (version 2022) software. Preparation of protein receptors including the removal of water molecules and original ligand sequences, as well as the usage of the “quick prep” option in MOE. Similarly, the preparation of the Quercetagetin ligand included applying the “wash” and “energy minimize” functions within the software. An induced-fit docking approach was employed, targeting all atoms during the docking process.

### Reagents and materials

Cell culture reagents, including minimum essential medium α (α-MEM, C12571500BT, Gibco), fetal bovine serum (FBS, 10099–141 C, Gibco), and penicillin/streptomycin (P/S, 15140122, Gibco), were all purchased from Gibco (Gaithersburg, MD, USA). Cytokines or chemicals used for osteoclast differentiation induction and treatment include macrophage colony-stimulating factor (M-CSF, 576406, Biolegend), receptor activator of nuclear factor-κB ligand (RANKL, 769406, Biolegend), lipopolysaccharide (LPS) (L8274, Sigma-Aldrich), N-Acetylcysteine (NAC) (HY-B0215, MCE) and Quercetagetin (V28848, Invivochem). Antibodies for western blotting (WB) were obtained from the following sources: anti-Akt (10176-2-AP), anti-Ctsk (11239-1-AP), anti-Keap1 (10503-2-AP) and anti-rabbit (SA00001-2) were from Proteintech (China); anti-Nrf2 (A0674) and anti-Nfatc1 (A1539) were from ABclonal (China), anti-phospho-Akt (4060) and anti-Pten (9559) were from Cell Signaling Technology (Danvers, MA, USA).

### In vitro cell culture and LPS stimulation

The procedure of cell culture was similar to our previous study [25]. In brief, bone marrow-derived monocytes (BMMs) were flushed out from the long bones of the biliteral hindlimbs from 8 to 12-week-old mice (C57BL/6). The isolated bone marrow cells were transferred to a complete medium (CM; α-MEM containing 10% FBS and 1% P/S) and cultured for 16 to 24 h. After red blood cell lysis, the nonadherent cells were collected and incubated in CM with M-CSF (20 ng/ml) at a density of 3 × 10^5^ cells/ml for 2 to 3 days to reach a 30%~50% confluence. BMMs were cultured with osteoclastogenesis induction medium (OIM) (CM with M-CSF (20 ng/ml) and RANKL (40 ng/ml)) for another 3 days to generate osteoclast precursors, and then RANKL was replaced by LPS (150 ng/ml) for following stimulation. For the treatment group and positive control, an indicated concentration of Quercetagetin and NAC was added to the CM from the onset of osteoclast differentiation induction.

### Cell viability assay

BMMs were cultured in a 96-well plate at a density of 3 × 10^5^ cells/ml and the cellular viability was detected by the Enhanced Cell Counting Kit 8 (CCK8, E-CK-A362, Elabscience) assay. Briefly, after the addition of indicated concentrations of Quercetagetin (1, 1.5, 2, 2.5, 3 µM) to each well containing osteoclast differentiation induction medium for 48 h stimulation, CCK-8 assay was performed according to the manufacturer’s instructions. Cells were then incubated at 37° for 2 h, and the absorbance at 450 nm wavelength was determined by a spectrophotometer (TECAN SPARK).

### Tartrate-Resistant alkaline phosphatase (TRAP) staining

The TRAP staining solution was manufactured based on Dr. Chevalier’s protocol with slight modifications [33, 34]. In brief, the Acetate-Tartrate buffer (AT buffer) was prepared in advance, composed of sodium acetate trihydrate (19 mg/ml), sodium tartrate trihydrate (150 µg/ml), and glacial acetic acid 100% (0.45%) in ultrapure water. Fast violet B salt (7 mg/ml) was then dissolved in the AT buffer and filtered through a 0.45 μm filter after thorough agitation. Naphthol solution was prepared with naphthol AS-TR phosphate disodium salt (2 mg/ml) dissolved in AT buffer. The successful fabrication of the TRAP staining solution was indicated by the emergence of yellow precipitates upon fully blending equal volumes of the violet and naphthol solutions. Mature osteoclasts were then fixed with 4% paraformaldehyde (PFA) for 15 min and stained with the TRAP solution. After 2 h incubation at 37°, the cells were rinsed with 4% sodium fluoride solution. The mature osteoclasts of each well were identified and quantified according to their TRAP-positive multinucleated nature with a minimum of three nuclei.

### F-Actin Phalloidin-iFluor staining

Mature osteoclasts were fixed with 4% PFA for 15 min at room temperature. Cells were incubated with Phalloidin-iFluor 594 reagent (ab176757, Abcam) at 37° for 2 h in the dark atmosphere. Cell nuclei were then stained with the DAPI solution. A confocal system (EVOS M700, Thermo Fisher Scientific) was employed to observe F-actin rings and nuclei of osteoclasts. The percentage of osteoclasts with F-actin rings was then calculated and recorded.

### Acridine orange (AO) staining

Mature osteoclasts were stained with AO solution (A6014, Sigma-Aldrich) according to the manufacturer’s instruction at 37℃ for 15 min. The acidic vesicles (red) and basic nuclei (green) were stained and observed with a confocal imaging system (Celldiscoverer 7, Zeiss), and the fluorescent intensity from red and green channels within the osteoclasts was recorded and assessed using the ZEISS ZEN 3.8 software. The intensity ratio of red/green was calculated to reflect the acid secretion function of osteoclasts.

### Bone resorption assay

BMMs were seeded in a bone resorption 48-well plate (CSR-BRA-48KIT; Cosmo Bio USA) with a density of 3 × 10^5^ cells/ml. The osteoclast induction process was generally consistent with introduced above under slight modification. Briefly, the replacement of RANKL to LPS was changed from day 3 to day 5, and LPS was employed for the osteoclastic induction on the last 3 days. On day 8, the culture medium was discarded and 5% sodium hypochloritesolution was added to the well plate. It was then rinsed with ddH_2_O for three times, and photographed after totally drying out under room temperature. The ImageJ software was then employed to analyze and quantify the resorption area of each well.

### Intracellular ROS measurement

Intracellular ROS level was determined by the H2DCFDA probe (S0033S, Beyotime) as described in the previous publication [35]. After cultured in the presence or absence of Quercetagetin for 24 h, osteoclast precursor cells were incubated with the DCFH-DA probe for 30 min at 37 °C. Following the removal of the DCFH-DA probe, RANKL (40 ng/ml), LPS (150 ng/ml), or a combination of LPS (150 ng/ml) and Quercetagetin (2 µM) were added to each sample group. Upon 10 min stimulation, the cells were examined using a confocal system (EVOS M700, Thermo Fisher Scientific) and analyzed with ZEISS ZEN 3.8 software.

### Western blotting (WB)

WB was performed based on our previous publication with slight modifications [36]. Cells were collected and lysed with RIPA buffer (R0020, Solarbio) containing 1% phosphatase inhibitor (CW2383; Cwbio) and 1% protease inhibitor (CW2200; Cwbio). BCA Protein Assay Kit (PC0020, Solarbio) was utilized to determine the protein concentration of each group. 30 µg heat-denatured proteins in each well were electrophoresed on an 8% SDS-page gel and transferred to a polyvinylidene fluoride (PVDF) membrane (0.22 μm). The membrane was then blocked by protein-free blocking buffer (PS108P, Epizyme) for 1 h and incubated with indicated primary antibodies at 4° overnight. HRP-conjugated goat anti-rabbit secondary antibody was used to conjugate protein bands by conjugating the primary antibody. The western ECL substrate (BioRAD) reacted blots were visualized with an imaging system (BioRAD) and analyzed by the Image J software.

### Quantitative Real-time polymerase chain reaction (qPCR)

The total RNA of cells was extracted by TRizol agent (9109, TaKaRa). The reverse transcription of total RNA into cDNA was performed using the RT Premix kit (AG11706, Accurate Biology) following the manufacturer’s protocol. The amplification and detection processes were performed employing the SYBR Green Premix kit (AG11701, Accurate Biology) and LightCycler 480II (Roche). GAPDH expression level was utilized as the endogenous control to analyze the relative expression of Acp5, Atp6vod2, Ctsk, Dc-stamp, Oc-stamp, Mmp9, Nfatc1, Tnfrfs11a and Traf6. All used primer sequences are displayed in Table 1.

**Table 1:** Primer sequences for quantitative Real-Time PCR

Target (GenBank accession no.)	Primers
Acp5 (NM 001102405.1)	F: ACCTTGGCAACGTCTCTGCAC
	R: GTCCAGCATAAAGATGGCCACA
Atp6v0d2 (NM_175406.3)	F: AACTCAGCAGGACTATGTCAACC
	R: CTTCTTCCTCATCTCCGTGTCAAT
Dcstamp (NM 029422.4)	F: TTCTCGTGTCAGTCTCCTTCTACC
	R: TTTCCCGTCAGCCTCTCTCAA
Ocstamp (NM_029021.1)	F: GTTCTGGACTTCATCCTCTTCGT
	R: GTGGTTGAGCCTGTGGTAGAT
Mmp9 (NM_013599.5)	F: GCCCTGGAACTCACACGACA
	R: TTGGAAACTCACACGCCAGAAG
Tnfrsf11a (NM_009399.5)	F: GCTTACCTGCCCAGTCTCATC
	R: AAGCATCATTGACCCAATTCCAC
Traf6 (NM_001303273.1)	F: AAAGCGAGAGATTCTTTCCCTG
	R: ACTGGGGACAATTCACTAGAGC
Nfatc1 (XM_036161029.1)	F: CCGTTGCTTCCAGAAAATAACA
	R: TGTGGGATGTGAACTCGGAA
Ctsk (NM_007802.4)	F: CTTCCAATACGTGCAGCAGA
	R: TCTTCAGGGCTTTCTCGTTC

### Collagen antibody-induced arthritis (CAIA) model

Eight-week-old DBA/1 male mice were purchased form Vital River Laboratory (Beijing, China), and randomly assigned into three groups (Control, CAIA, CAIA + Quercetagetin). All mice were fed under the specific pathogen-free (SPF) conditions (22 ℃) with 12 h light/dark cycle and freely accessed SPF–standard food and water. Arthritogenic Monoclonal Antibody Cocktail Kit (Chondrex, 53010) was employed to construct CAIA model following manufacture’s instruction. Briefly, mice in the CAIA and CAIA + Quercetagetin groups received 150 mg antibody cocktail on day 0 and 50 µg LPS on day 3 through intraperitoneal (IP) injection. From day 5, mice in the CAIA + Quercetagetin group received 40 mg/kg Quercetagetin through IP injection every day until sacrificed. The arthritis score was recorded from day 5 to day 13 based on previous study [37]. The arthritis score criteria is presented as follows: 0 = no evidence of erythema or swelling; 1 = erythema and mild swelling confined to the tarsals or ankle joint; 2 = erythema and mild swelling extending from the ankle to the tarsals; 3 = erythema and moderate swelling extending from the ankle to metatarsal joints; and 4 = erythema and severe swelling encompassing the ankle, paw and digits, or ankylosis of the limb [38].

### Micro-Computed tomography (Micro-CT) analysis

The hinder limbs of mice were fixed in 4% PFA for 24 h, then rinsed and storage in 70% ethanol. The feet and ankle joints were scanned by Micro-CT (Quantum GX2) with parameters of 90KV, 88µA, and the two-dimensional (2D) sectional figures and three-dimensional (3D) reconstruction images were obtained. Bone mineral density (BMD) of ankle joints were calculated with Analyze12 software (AnalyzeDirect).

### Statistical analysis

All in vitro experiments were performed using biologically independent triplicates, whereas in vivo validations utilized five distinct biological samples. GraphPad Prism 9.4 software was used for data analysis, and data were presented as the mean ± standard deviation (SD). T-test or one-way ANOVA was conducted for the analysis between two groups or multiple groups’ comparison. *P* < 0.05 was considered as statistical significance.

## Results

### Identification of bioactive compounds in Flos eriocauli and their potential targets on RA

**Fig. 1:**
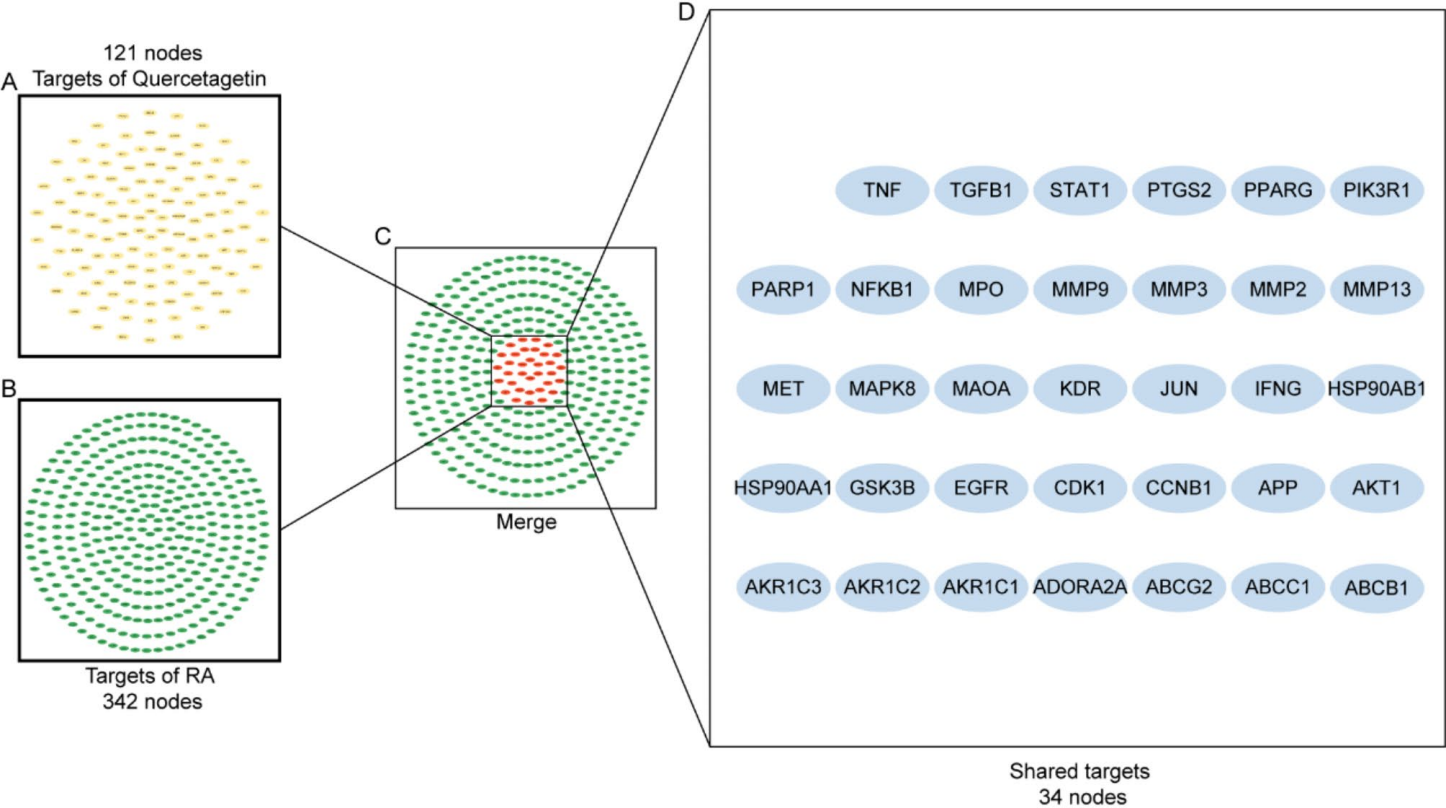
Identification of shared gene targets between Quercetagetin and rheumatoid arthritis (RA). (**A**) 121 predicted targets of Quercetagetin. (**B**) 342 potential targets associated with RA. (**C**) Circular diagram outlined the (**D**) common targets related to Quercetagetin and RA, highlighting 34 shared targets

To explore the constituents of Flos eriocauli and elucidate their potential targets on RA, a total of 24 bioactive compounds presented in Flos Eriocauli were extracted from the TCMSP database as displayed in Table S1. Based on numerous existing publications, OB ≥ 30% and DL ≥ 0.18 are widely accepted as pivotal parameters to assess the translational and application potential of natural compounds [39, 40]. Additionally, the impermeability to BBB could minimize unwarranted interference with the cerebrovascular system, with a parameter of BBB<-0.6 serving as a suitable parameter [41]. Therefore, upon the settings for screening parameters that include OB ≥ 30%, DL ≥ 0.18, BBB<-0.6, Quercetagetin and two other bioactive compounds emerged, with Quercetagetin displaying superior positioning when ranked by BBB (See Table 2). Thereafter, by utilizing the TCMSP, HERB, and SwissTarget databases, 121 targets that related to Quercetagetin were spotted (Table S2). For the identification of RA-related targets, the term “rheumatoid arthritis” was employed for searching in the CTD database. Considering a relatively high amounts of RA-related targets in the database, an inference score greater than 50 was set to narrow the retrieved results from CTD. This process identified a total of 342 targets as RA-related targets (Table S3). Subsequently, the intersection of the results from potential Quercetagetin targets and RA-related targets yielded 34 shared targets, which were adopted for further use (Fig. 1), especially in the functional analysis.

**Table 2:** Three superior bioactive compounds from Flos eriocauli

Molecule Name	MW	AlogP	Hdon	Hacc	OB (%)	Caco-2	BBB	DL	FASA-	HL
Quercetagetin	318.3	1.24	6	8	45.01	-0.06	-0.93	0.31	0.34	13.82
Patuletin	332.3	1.49	5	8	53.11	0.01	-0.71	0.34	0	14.31
Quercetin	302.3	1.5	5	7	46.43	0.05	-0.77	0.28	0.38	14.4

### Quercetagetin-RA target network and analysis

**Fig. 2:**
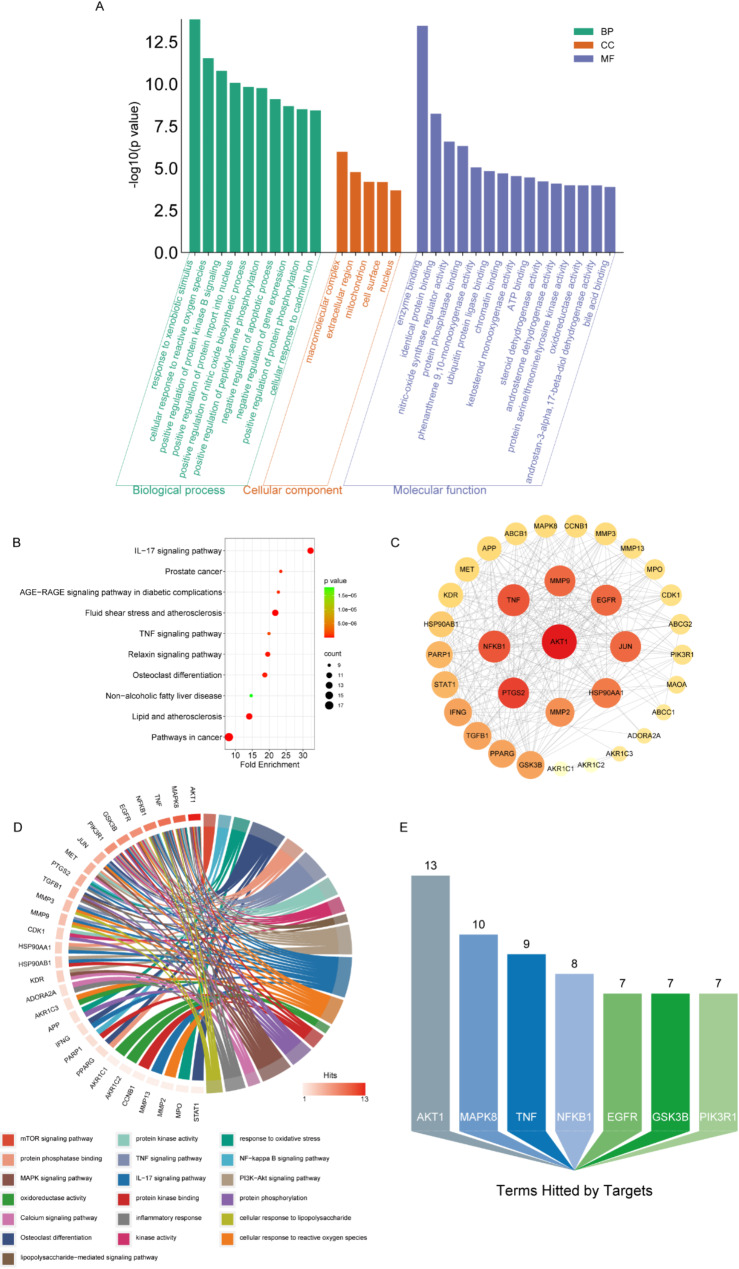
Quercetagetin-RA target network and analysis. (**A**) Histogram of Gene Ontology (GO) enrichment analysis displays the higher-rank terms of biological process, cell component, and molecular function, according to the 34 shared targets. (**B**) Bubble chart of KEGG pathway analysis corresponding to the shared targets. The top 10 enriched pathways are shown. (**C**) The Protein-Protein Interaction (PPI) and (**D**) chord diagram illustrate the network of relationships between putative targets and terms related to RA. (**E**) Quantification of terms hitted by targets in (**D**)

Next, to investigate the possible mechanisms of Quercetagetin in the treatment of RA, the GO and KEGG enrichment analyses were then conducted. By uploading the 34 shared targets to David (v6.8) (https://david.ncifcrf.gov/home.jsp) [30], a total of 417 GO and KEGG terms were significantly enriched (*P*<0.05), including 206 biological process (BP) terms, 31 cellular component (CC) terms, 67 molecular function (MF) terms, and 113 pathway terms (Table S4). The most significantly enriched terms for BP, CC, MF, and KEGG are displayed (Fig. 2A-B) and suggested an intimate correlation of Quercetagetin with osteoclast differentiation, ROS response, and inflammatory process. A PPI network was further constructed through the STRING database using the same 34 shared targets, during which, the minimum interaction score was defined at 0.4, and disconnected proteins were excluded. To further analyze and visualize the results from the STRING database (Table S5), Cytoscape software was utilized to calculate the degree and radiality among these targets (Fig. 2C). The results implied that AKT1, PTGS2, NFKB1, and TNF may act as interacting hubs among these proteins. Moreover, their identifications suggested that Quercetagetin may treat RA through pluralism pathways and various targets. To further elucidate the connection between shared targets and RA or osteoclast-related terms, a target-term interaction network was constructed and measured (Fig. 2D-E). Upon a comprehensive analysis of all bioinformatics results, we established AKT1, TNF, PTGS2, and NFKB1 as potential core targets of Quercetagetin treatment given their significant associations with diverse enrichment terms.

### Revelation of potential action mode of Quercetagetin in RA-related targets

**Fig. 3:**
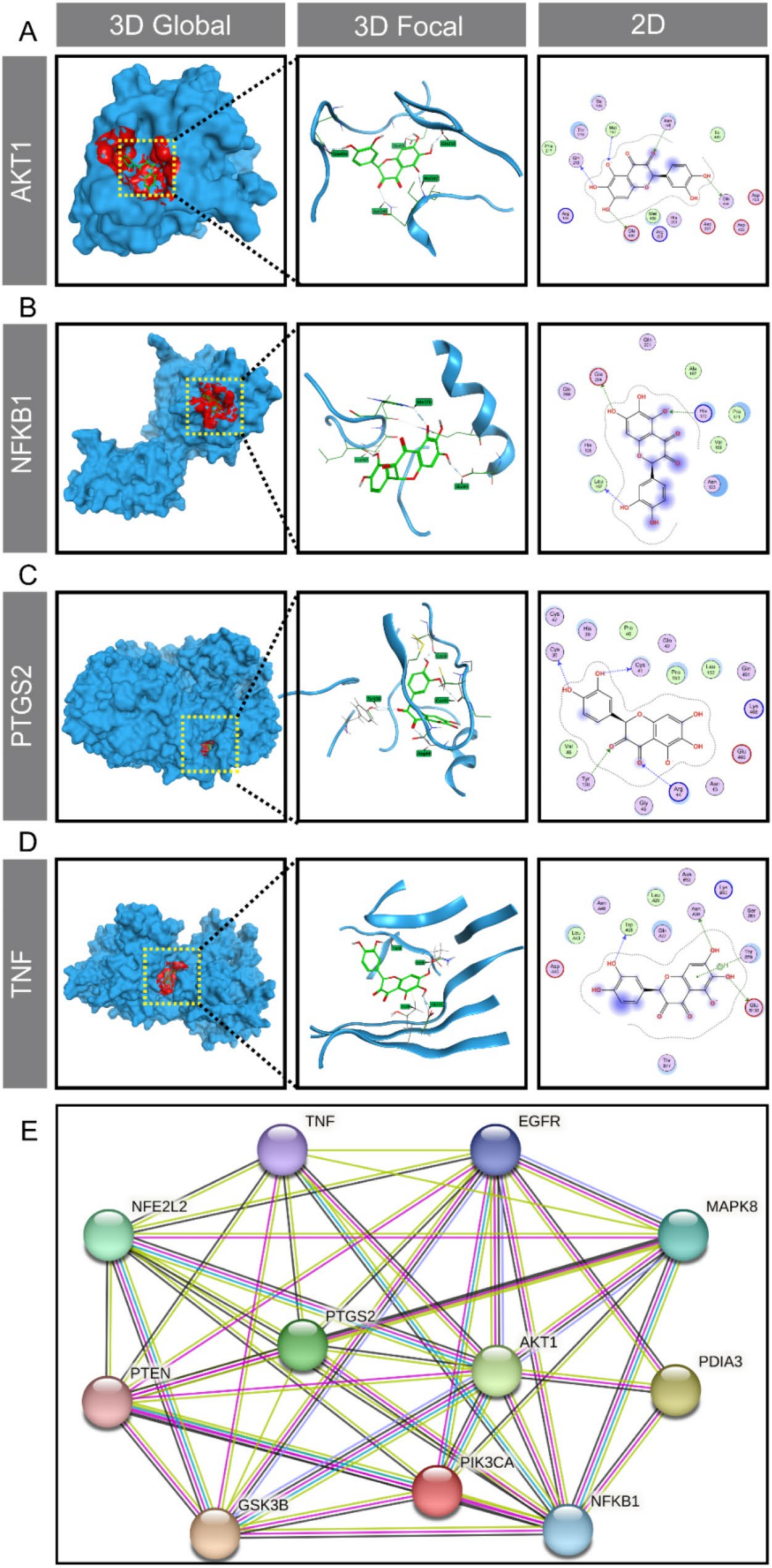
Revelation of potential action mode of Quercetagetin in RA-related targets. (**A**-**D**) The molecular docking mode of Quercetagetin with AKT1, NFKB1, PTGS2 and TNF shown in 3D or 2D model. (**E**) The Protein-Protein Interaction (PPI) network reveals relationships of highly interactive and related targets

To confirm the binding mode of Quercetagetin with these potential core targets, molecular docking was conducted, primarily through hydrogen (H) bond, ionic bond, or H-pi bond interactions. The results showed that Quercetagetin could successfully bond with multiple sites of the targeted proteins, including ASP, ASN, MET residues of AKT1 (Fig. 3A), ARG, LYS residues of NFKB1 (Fig. 3B), ARG, GLY residues of PTGS2 (Fig. 3C), as well as ARG, GLU, LEU residues of TNF (Fig. 3D). The binding energy was subsequently calculated to demonstrate the affinity between the ligand and receptor. As illustrated in Table 3, the binding energies between Quercetagetin and AKT1, NFKB1, PTGS2, and TNF are − 5.9, -5.6, -5.4, and − 5.7 respectively. These energy levels indicate a strong binding affinity of the proteins interacting with Quercetagetin, and the highest possibility was shown on AKT1. To better illustrate the relationship between core targets and shared targets, as well as their connection with the key regulators of signaling pathways from our bioinformatics results, we have reconstructed the diagram of direct action of these proteins through PPI analysis. The result demonstrated a compelling correlation between the core targets, Pten/AKT and Nrf2 signalings, as well as the involvement of ERp57 (PDIA3) regulatory signaling (Fig. 3E). Since Pten/PI3K/AKT signaling, Nrf2 signaling and ERp57/calcium signaling are reported to be canonical pathways in osteoclast differentiation and significantly influence osteoclast behaviors under various pathological conditions [42–44] our results, therefore, depict a strong correlation between the therapeutic mechanisms of Quercetagetin in RA and the impact on inflammatory osteolysis.

**Table 3:** Docking results of Quercetagetin with key targets

Target	Binding energy/(kcal/mol)	Binding residues
AKT1	-5.9	ASP, ASN, MET
NFKB1	-5.6	ARG, LYS
PTGS2	-5.4	ARG, GLY
TNF	-5.7	ARG, GLU, LEU

### Quercetagetin impairs RANKL or LPS-induced osteoclastogenesis

**Fig. 4:**
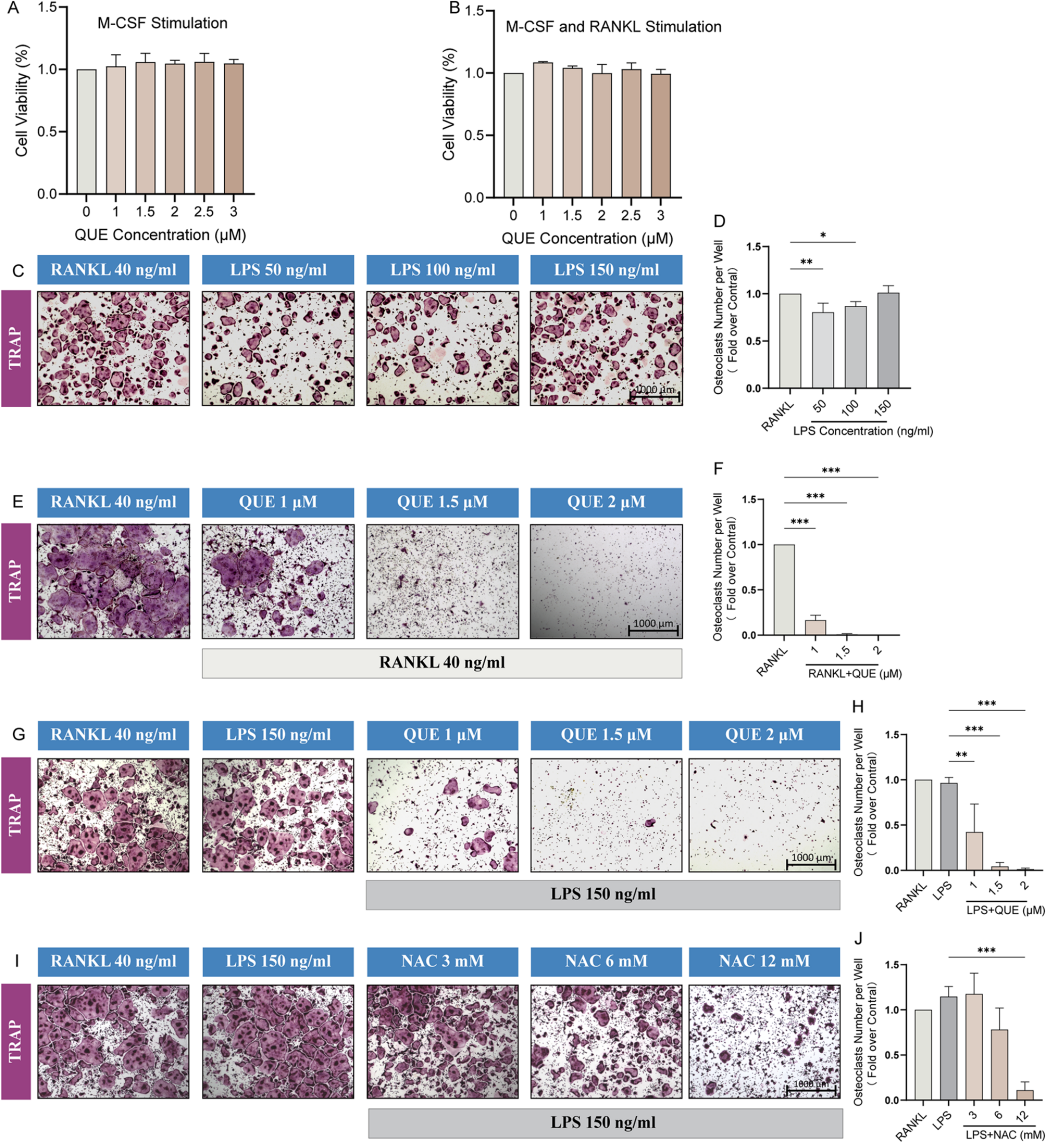
Quercetagetin impairs RANKL or LPS-induced osteoclastogenesis. (**A**-**B**) CCK-8 assay for cell viability test. Bone marrow-derived monocytes (BMMs) were stimulated with Quercetagetin (0, 1, 1.5, 2, 2.5, and 3 µM) in complete medium with M-CSF (20 ng/ml) only (**A**) or with M-CSF (20 ng/ml) and RANKL (40 ng/ml) (**B**) for a period of 48 h. (**C**) TRAP staining of osteoclasts under different concentrations of LPS. BMMs were stimulated with osteoclastogenesis induction medium (OIM) (comprising CM with M-CSF and RANKL) for a 5-day period for full differentiation, or initially pretreated with OIM for 60 ~ 72 h, after which the RANKL was replaced with LPS (50, 100, and 150 ng/mL) for the subsequent stimulation. (**D**) Quantitative analysis of TRAP-positive cells with more than three nucleis (TRAP + MNCs) in (**C**). (**E**, **G**, **I**) TRAP staining of osteoclasts differentiated from BMMs under various treatments of Quercetagetin and NAC. BMMs were induced with OIM for a full 5-day osteoclast differentiation with different concentrations of Quercetagetin (1, 1.5 and 2 µM), or initially stimulated with OIM and Quercetagetin or NAC (3, 6, 12mM) for a 60 ~ 72 h pretreatment to generate pre-osteoclasts, after which the RANKL was replaced with LPS to induce the remaining differentiation. (**F**, **H**, **J**) Quantitative analysis of TRAP + MNCs in (**E**), (**G**) and (**I**). All quantitative data were presented as mean ± SD from three biologically independent experiments. *** *p* < 0.001, ** *p* < 0.01, * *p* < 0.05. QUE: Quercetagetin, NAC: N-Acetylcysteine

Considering the established relevance of Quercetagetin with osteoclast-related pathways as detailed above, we next explored its role in LPS-induced osteoclastogenesis, recognized as classical in vitro model representing inflammatory bone erosion in RA [45, 46]. To examine the actual effect of Quercetagetin on osteoclast differentiation under RA conditions as hypothesized, the CCK-8 assay was first applied to determine the viability of osteoclast precursor cells after a 48 h OIM stimulation (with or without RANKL) in the presence of indicated concentrations of Quercetagetin (1, 1.5, 2, 2.5, 3 µM). The results demonstrated no obvious influence on cellular activities of Quercetagetin within the set concentrations (Fig. 4A and B), thereby establishing a minimum safe threshold for subsequent biological validations.

Concurrently, to ascertain the optimal dose of LPS for osteoclast induction, BMMs were initially subjected to OIM (in the presence of RANKL) for 60 to 72 h culturation to generate sufficient pre-osteoclasts as described in the previous study [34]. Subsequently, RANKL was replaced with indicated concentrations of LPS (50, 100, and 150 ng/ml) for further cultivation. After a 5-day complete osteoclast differentiation, TRAP staining was conducted to quantify mature osteoclasts. For these pre-osteoclasts pretreated by RANKL, a dose-dependent response to LPS stimulation was observed, with a concentration of 150 ng/ml LPS exhibiting the comparable effect to solely RANKL induction (Fig. 4C and D). Next, pre-osteoclasts continuously stimulated with RANKL or replaced by LPS, were separately treated with Quercetagetin. A dose-dependent inhibitory effect was demonstrated in both groups, with a significant impediment in osteoclastogenesis observed at a concentration of 2 µM, which was therefore chosen for the subsequent experiments (Fig. 4E-H). Meanwhile, we incorporated NAC, a widely recognized inhibitor of osteoclast differentiation known for its role in scavenging the ROS, as a positive control group in our study. Notably, the suppressive effect of NAC on osteoclastogenesis is essentially consistent with the Quercetagetin treatment group (Fig. 4I-J).

### Quercetagetin suppresses the resorption function of osteoclasts

**Fig. 5:**
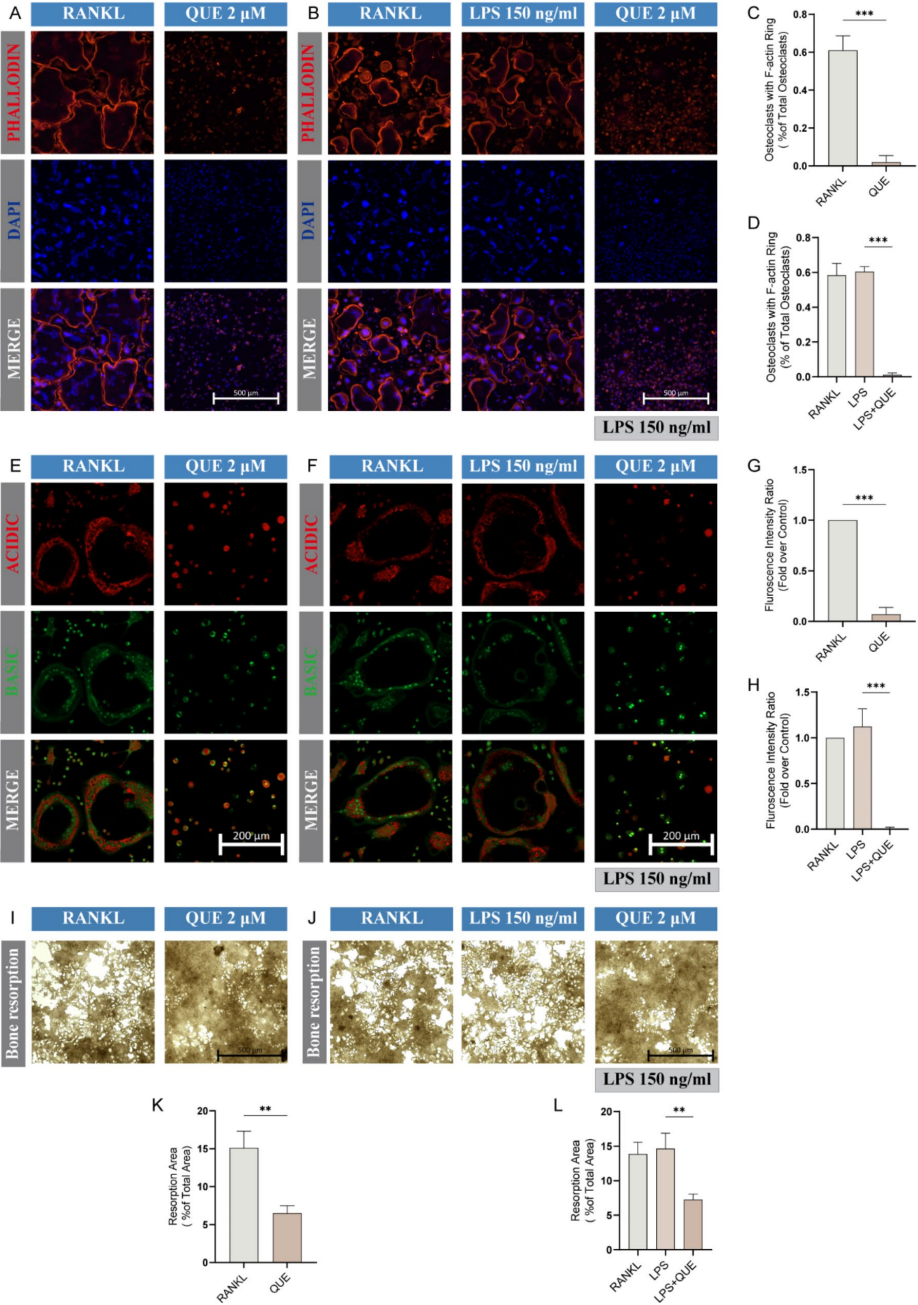
Quercetagetin suppresses the resorption function of osteoclasts. (**A**, **B**) Effect of Quercetagetin on F-actin ring formation. Cell culture was performed as described in Fig. 4, and the F-actin rings (red) and nucleus (blue) were captured utilizing a confocal microscope after staining with phalloidin-iflour 594 and DAPI. (**C**, **D**) Quantitative analysis of the proportion of osteoclasts displaying intact F-actin rings in (**A**) and (**B**). (**E**, **F**) The effect of Quercetagetin on the acid secretion ability of osteoclasts. Acridine orange (AO) staining was performed to visualize the acidic vesicles (red) of osteoclasts. (**G**, **H**) Quantitative analysis of the fluorescence intensity ratio (red to green) of osteoclasts as shown in (**E**) and (**F**). (**I**, **J**) Bone resorption assay to determine the resorptive ability of osteoclasts treated with Quercetagetin. (**K**, **L**) Quantitative analysis of the resorption area in (**I**) and (**J**). All quantitative data were presented as mean ± SD from three biologically independent experiments. *** *p* < 0.001, ** *p* < 0.01, * *p* < 0.05. QUE: Quercetagetin

We next explored the suppressive impact of Quercetagetin on osteoclast function. The formation of F-actin rings is necessary for osteoclasts to exert resorption function [35]. Visualized by phalloidin staining, we discerned approximately 60% of the osteoclasts with intact F-actin rings in either the RANKL or LPS groups. In contrast, the Quercetagetin-treated group displayed a dramatic shrinkage in this proportion, regardless of LPS or RANKL stimulation (Fig. 5A-D). Likewise, AO dye, which emits red fluorescence under acidic conditions, is used to visualize the acid vesicles of osteoclasts [35]. Notably, there was a significant decline in the red-to-green fluorescence intensity ratio amongst osteoclasts in the Quercetagetin group (Fig. 5E-H), demonstrating repression of acid secretion in osteoclasts. To directly verify the bone resorption activity of osteoclast in each group, a bone resorption assay was performed with hydroxyapatite-coated 48-well plate, verifying an impaired bone resorption function of osteoclast after Quercetagetin treatment (Fig. 5I-L).

### Quercetagetin inhibits the expression of osteoclastic marker genes and proteins

**Fig. 6:**
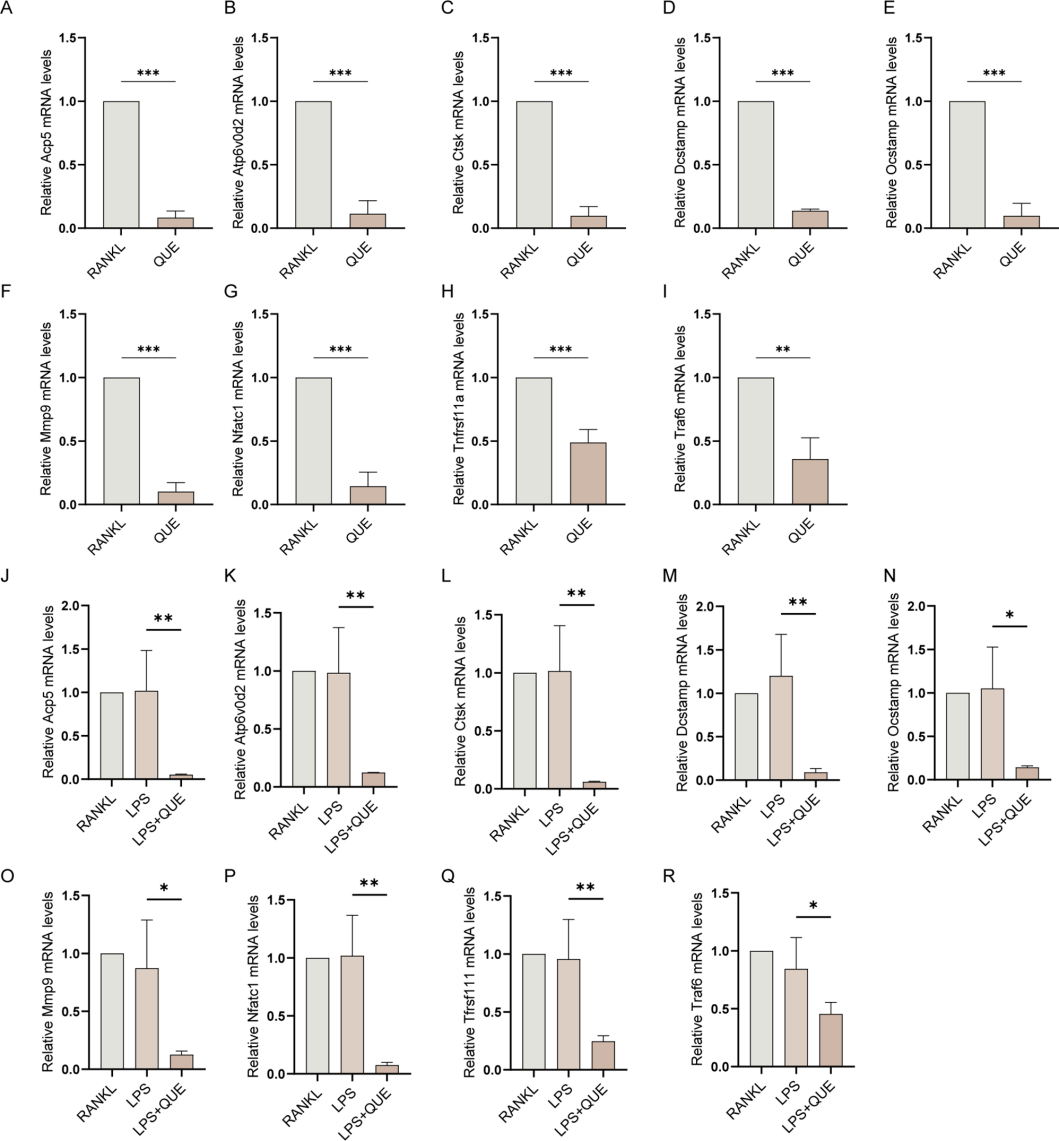
Quercetagetin inhibits the expression of osteoclastic marker genes. RT-qPCR analysis showed mRNA levels of Acp5 (**A**, **J**), Atp6v0d2 (**B**, **K**), Ctsk (**C**, **L**), Dcstamp (**D**, **M**), Ocstamp (**E**, **N**), Mmp9 (**F**, **O**), Nfatc1 (**G**, **P**), Tnfrsf11a (**H**, **Q**), and Traf6 (**I**, **R**). Quantitative results were normalized to Gapdh with a 2^−ΔΔ^Ct method. All quantitative data were presented as mean ± SD from three biologically independent experiments. *** *p* < 0.001, ** *p* < 0.01, * *p* < 0.05. QUE: Quercetagetin

**Fig. 7:**
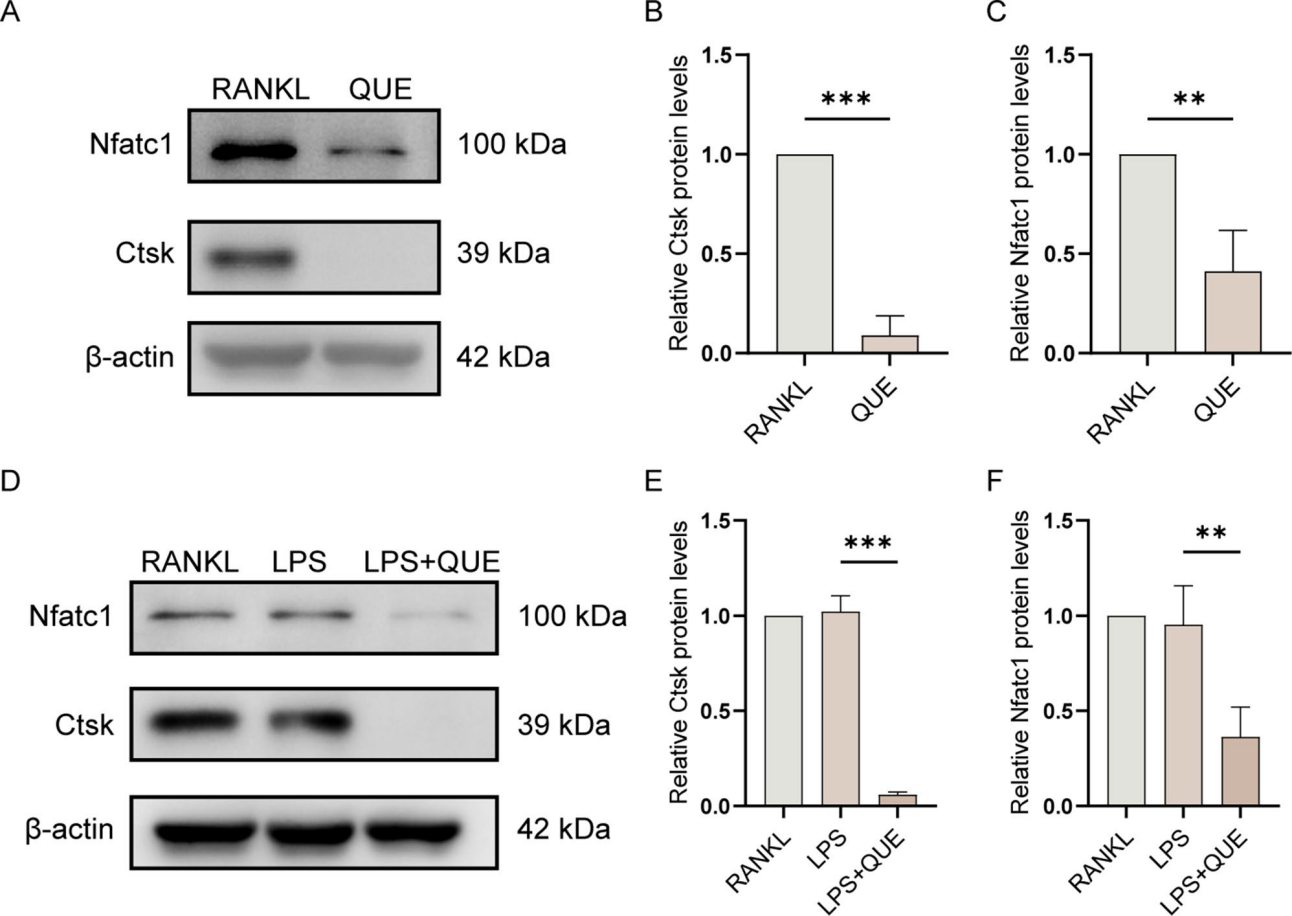
Quercetagetin inhibits the expression of osteoclastic marker proteins. (**A**, **D**) WB assay showed the protein expression level of Nfatc1 and Ctsk in osteoclasts post various cultures. The quantitative data were normalized to β-actin and presented as mean ± SD in (**B**, **C**, **E**, and **F**). All data were obtained from three biologically independent experiments. *** *p* < 0.001, ** *p* < 0.01, * *p* < 0.05. QUE: Quercetagetin

To further unveil the molecular mechanisms responsible for Quercetagetin’s suppressive effect on LPS-induced osteoclast behaviors, we assessed the expression of canonical osteoclast markers from both the transcriptional and translational levels. QPCR analysis revealed that all osteoclast-specific genes, including those related to osteoclastic differentiation (Traf6, Tnfrsf11a, and Nfatc1), pre-osteoclast fusion (Atp6v0d2, Dcstamp, and Ocstamp), and bone resorption function (Ctsk, Acp5 and Mmp9), presented comparable or even escalated expression level in the LPS group compared to the RANKL group, but were markedly downregulated under Quercetagetin treatment, regardless of LPS or RANKL stimulation (Fig. 6). We further corroborated these observations through WB assay at the protein level, which yielded comparable results. A significant reduction of Nfatc1 and Ctsk protein expression was observed in the Quercetagetin-treated conditions (Fig. 7), aligning with our previous observations that Quercetagetin inhibited osteoclast formation and function (Figs. 4 and 5).

### Quercetagetin suppresses LPS-induced osteoclastogenesis by activating the Nrf2-ROS scavenger system and repressing the Pten/AKT signaling pathway

**Fig. 8:**
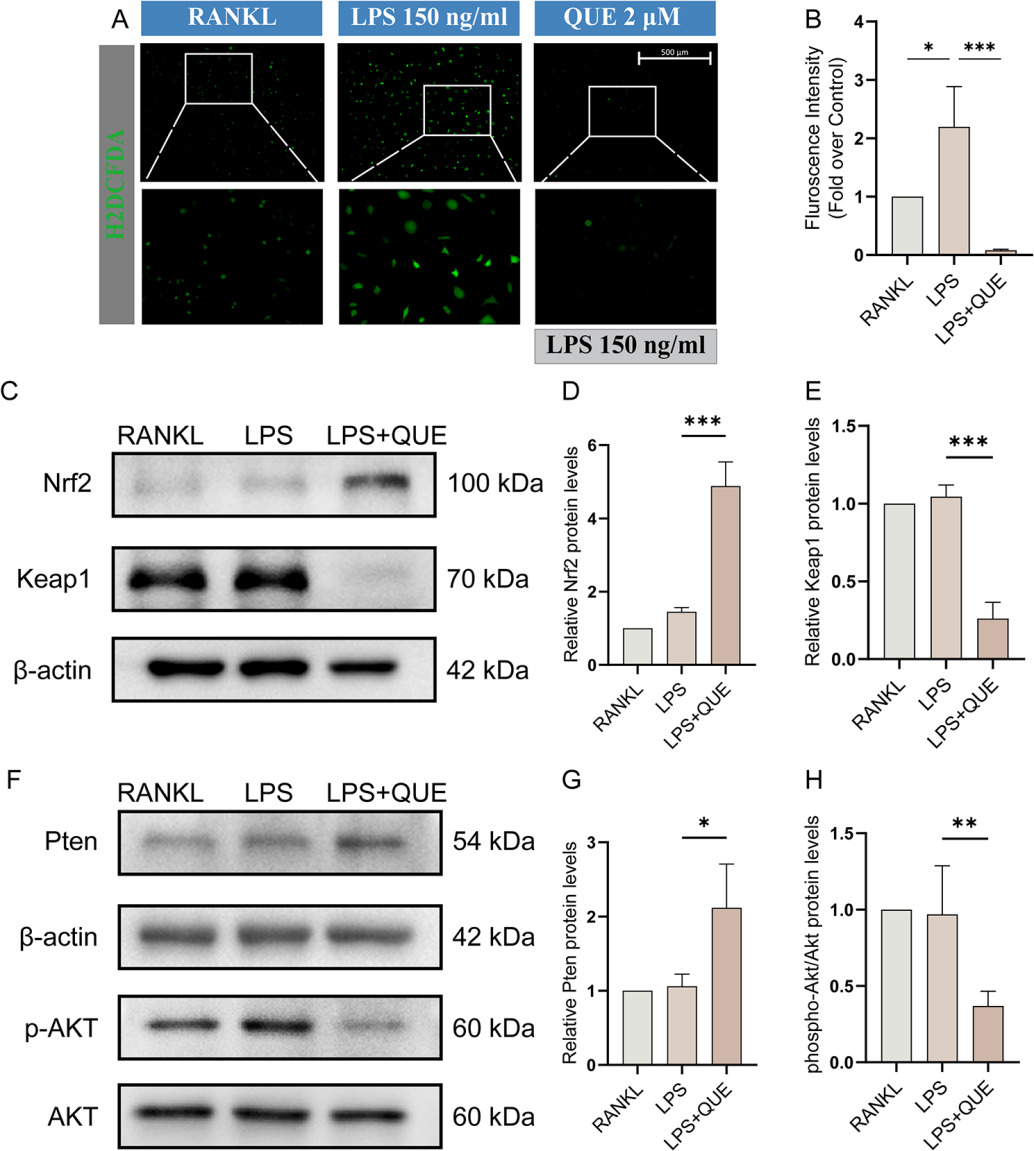
Quercetagetin suppresses LPS-induced osteoclastogenesis by activating the Nrf2-ROS scavenger system and repressing the Pten/AKT signaling pathway. (**A**) Intracellular ROS level of osteoclasts precursors. Followed by culturation with or without Quercetagetin (2 µM) for 24 h, osteoclast precursor cells in each group were treated with DCFH-DA probe for 20 min and stimulated with RANKL (40 ng/ml), LPS (150 ng/ml), or LPS and Quercetagetin (2 µM) for 10 min. Intracellular ROS was labeled by DCFH-DA probe and visualized by a three-dimensional digital confocal system (EVOS M700, Thermo Fisher Scientific). (**B**) Quantitative analysis of the fluorescence intensity in (**A**). (**C**, **F**) WB showed the protein expression level of Nrf2, Keap1, Pten, p-AKT and AKT in the groups treated with RANKL, LPS, and LPS + Quercetagetin. Protein of each group was extracted 4 days after first RANKL addition. Protein quantification of Nrf2, Keap1 and Pten was normalized to β-actin and shown in (**D**, **E**, and **G**). (**H**) quantitative analysis of p-AKT/AKT level in (**F**). All quantitative data were presented as mean ± SD from three biologically independent experiments. *** *p* < 0.001, ** *p* < 0.01, * *p* < 0.05. QUE: Quercetagetin

From our bioinformatics results, Pten/PI3K/AKT signaling and Nrf2 signaling may play key roles in the therapeutic mechanisms of Quercetagetin in RA, particularly concerning inflammatory osteolysis. ROS is widely recognized as central mediators in the aforementioned inflammatory-related pathways, and serves as a significant contributor to osteoclast regulation [42, 47] Therefore, we proceeded to investigate the potential mechanisms through which Quercetagetin, by modulating ROS-associated signaling, impedes LPS-induced osteoclast differentiation and function. After receiving indicated stimulation, the intracellular ROS level of osteoclast precursor cells was detected by the H_2_DCFDA probe. Compared with the RANKL group, LPS triggered a substantial escalation in ROS generation. However, Quercetagetin curbed this surge, managing to restrict the ROS level to a considerably lower quantity (Fig. 8A and B). To authenticate the molecular mechanism explaining this phenomenon, we conducted a WB assay to identify variations in the ROS scavenger system. The results revealed that LPS stimulation mildly provoked the expression of Nrf2 protein, likely a response to ROS overproduction. Notably, Quercetagetin treatment markedly fostered Nrf2 expression while simultaneously reducing Keap1 expression (Fig. 8C-E). A significant promotion of Pten expression and repression of AKT phosphorylation were also observed after Quercetagetin treatment (Fig. 8F-H). Collectively, these findings underscore the potential effectiveness of Quercetagetin in fine-tuning the ROS regulation system and the Pten/AKT/Nfatc1 axis, which ultimately contribute to the inhibition of LPS-induced osteoclast differentiation and function.

### Quercetagetin alleviates arthritis progression and bone destruction in CAIA model

**Fig. 9:**
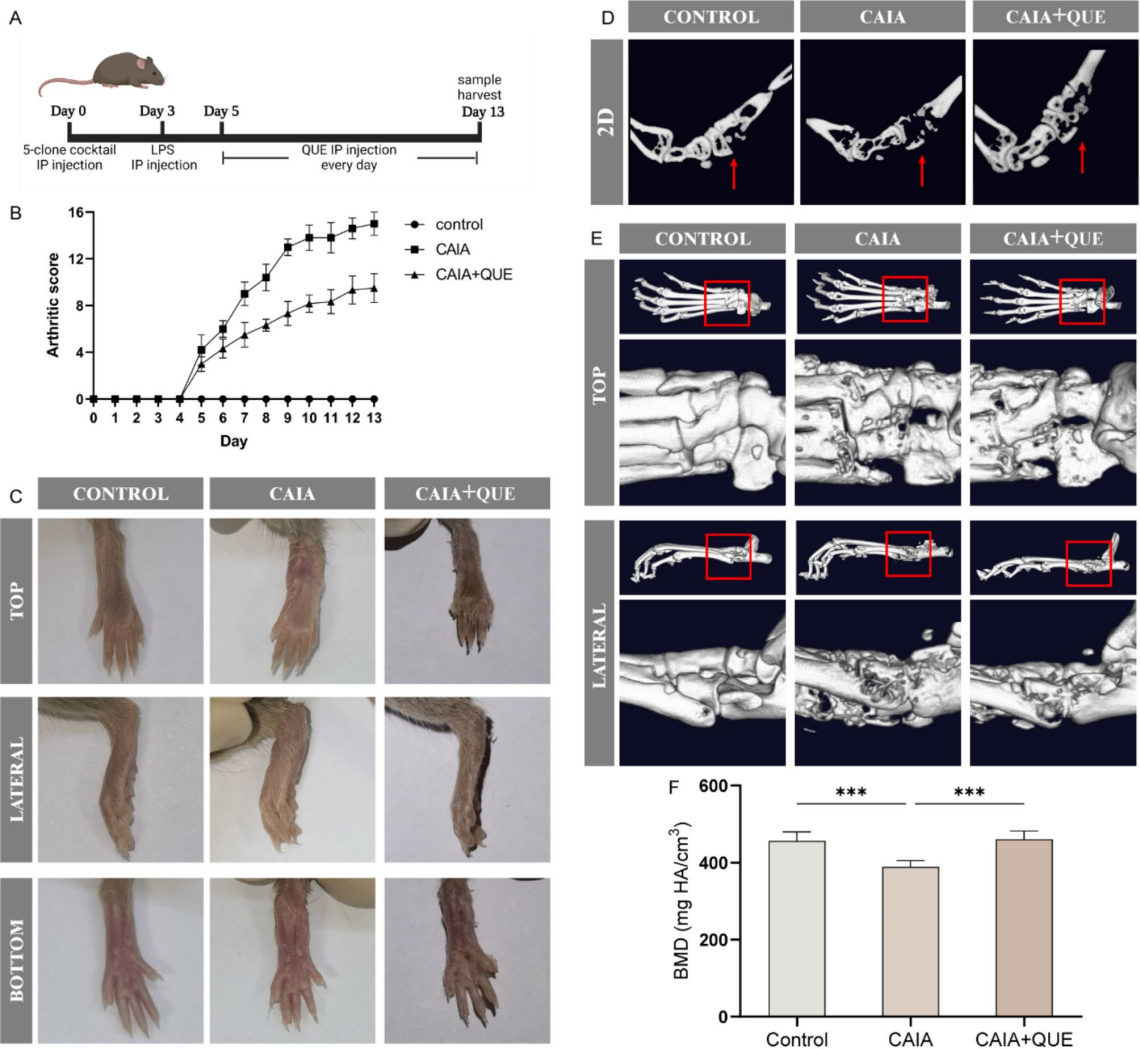
Quercetagetin alleviates arthritis progression and bone destruction in CAIA mice. (**A**) Illustration of CAIA model establishment and Quercetagetin injection strategy. (**B**) Arthritis score of mice in control, CAIA, and CAIA + Quercetagetin groups from day 5 to day 13. (**C**) The photographs of hind paws of mice were captured on day 13. (**D**-**E**) Two-dimensional sectional (2D) and three-dimensional (3D) reconstruction images of the hind paws in three different groups. (**F**) Bone mineral density (BMD) of the ankle joints of mice. All quantitative data were presented as mean ± SD. *n* = 5, *** *p* < 0.001, ** *p* < 0.01, * *p* < 0.05. QUE: Quercetagetin

Based on the bioinformatic evidence and results obtained from in vitro verification, we further validated the potential effect of Quercetagetin on CAIA mice. Mice were randomly assigned into control, CAIA and CAIA + Quercetagetin group, and injected with Quercetagetin (40 mg/kg) or saline every day through IP injection from day 5 to day 13 (Fig. 9A). The inflammation and redness of mice hind paws were obviously noticed in CAIA group after 8 days of injection, and were greatly attenuated after Quercetagetin treatment (Fig. 9C). The arthritis score shown a maximum difference of 5 points between CAIA and CAIA + Quercetagetin group on day 13 (Fig. 9B-C). 2D sectional figures and 3D reconstruction images of feet and ankle joint revealed the bone destruction in CAIA group, and consistent results were also presented on the BMD values of ankle joints. In contrast, Quercetagetin treatment significantly protect the small joints and prevent bone destruction (Fig. 9B-F). Therefore, our in vivo assay confirmed the therapeutic potential of Quercetagetin in mitigating RA-related inflammatory osteolysis.

## Discussion

The exploration for effective therapeutic agents to alleviate RA articular symptoms and retard disease progression remains facing tremendous challenges. Quercetagetin, a flavonoid compound extracted from Flos eriocauli, has demonstrated anti-inflammation and anti-virus properties in previous studies [48, 49]. In this study, by using network pharmacological methods, we postulate a correlation exists between Quercetagetin and RA, with Quercetagetin likely playing a pivotal role in controlling both the inflammatory process and osteoclast differentiation-related pathways. Intriguingly, our biological validations provide initial evidence of Quercetagetin’s potent inhibitory effect on osteoclast differentiation and function post LPS stimulation, at both cellular and molecular levels. This effect can potentially be ascribed to its regulatory function on ROS metabolism via Nrf2 signaling and Pten/AKT/Nfatc1 axis.

The identical targets between Quercetagetin and RA were firstly interpreted in this study, aiming to predict the potential pharmacodynamic mechanisms of Quercetagetin in RA treatment. Interestingly, 34 shared targets were presented to be involved in the regulation of the inflammatory process and osteoclast differentiation during RA. Prior research indicates that osteoclast, the primary bone-resorbing cell, are susceptible to various inflammatory factors and elevated ROS levels [50, 51]. This suggests that inflammation-induced osteoclast hyperactivity, observed in pathological osteolysis of RA, serves dual roles– as a central mediator and a crucial intervention target [45, 52]. Flavonoid compounds represent a broad class of natural products with significant anti-inflammatory, anti-oxidant, and immune regulation properties, and have thus become an important treatment option for RA [22]. Belonging to the flavonoid family, Quercetagetin is expected to possess the common characteristics including anti-inflammatory or even anti-osteoclast capabilities. Furthermore, core targets associated with Quercetagetin displayed higher interaction relationship with other protein targets, demonstrated a broad involvement in inflammatory process and osteoclast differentiation, and possessed excellent docking modes and scores with Quercetagetin. Therefore, the network pharmacological and molecular docking results raised Quercetagetin as a promising candidate for the prevention or treatment of RA-induced bone erosion.

We further validated the biological effect of Quercetagetin on osteoclast behaviors under LPS stimulation. LPS is recognized as an inducer of the inflammatory response, initiating inflammatory osteolysis and other disorders [53]. Through activating either surface or intracellular receptors of macrophages and other host cells, LPS can activate the inflammasome-related response, bolstering the production of inflammatory factors, such as IL-1β, IL-6, 1 L-8, and TNFα, thereby facilitating the activity of osteoclast lineage cells [54]. However, LPS stimulation alone could not dominate osteoclast differentiation from primary BMMs. Instead, it plays a crucial role as an inducer for the initial fusion process of pre-osteoclasts, alongside the involvement of RANKL. This facilitates the secretion of inflammatory factors by macrophages, which, under LPS stimulation, promotes further osteoclast maturation and function, regardless of whether RANKL removal occurs post the cell fusion stage [55]. We found corroborating results in our previous study as well, demonstrating that LPS induced osteoclast differentiation in a dose-dependent manner upon RANKL withdrawal, and a similar effect to RANKL stimulation was observed with 150 ng/ml of LPS. Nonetheless, Quercetagetin treatment led to a significant decrease in mature osteoclast number, regardless of LPS or RANKL stimulation. Identified by other researchers and our team, NAC could inhibit RANKL, LPS and dexamethasone-induced osteoclastogenesis, as well as LPS-induced osteolysis through the activation of the antioxidant system and scavenging ROS [35, 36, 55]. Therefore, we introduced NAC as a positive control in this study, which exhibited a dose-dependent inhibitory effect on osteoclast differentiation, closely mirroring the effect observed in the Quercetagetin treatment group.

Validations in osteoclast functional assays obtained similar results. The formation of the F-actin ring is critical for osteoclast adhesion and motility, which creates a locally sealed zone to secret inner acidic vesicles, thereby facilitating bone resorption [56]. Our study confirmed comparable effects of RANKL and LPS in promoting F-actin rings and acidic compartment formation, which were both significantly repressed by Quercetagetin. Besides, the bone resorption assay directly proved an impaired bone resorption function of osteoclasts treated with Quercetagetin. Therefore, these cellular phenotypic results imply that Quercetagetin may serve as an effective inhibitory agent in affecting osteoclast activities.

These changes in cell behaviors are phenotypic reflections of the alterations in the expression of marker genes and proteins. Our research demonstrated that Quercetagetin repressed the expression of osteoclast-specific genes involved in osteoclastic differentiation (Traf6, Tnfrsf11a, and Nfatc1), osteoclast fusion (Atp6v0d2, Dcstamp, and Ocstamp), and bone resorption function (MMP9, Ctsk and Acp5) post both LPS and RANKL stimulation. This inhibitory effect of Quercetagetin is confirmed at both the transcriptional and translational levels. In vivo assays also offer stronger evidence during treatment-effect verification. In our study, the CAIA model was established to verify the truly effect of Quercetagetin in RA condition. As expected, Quercetagetin successfully alleviated joint erythema and swelling, and retarded bone destruction in CAIA mice. Therefore, the consistence results obtained from bioinformatics analysis, in vitro assay and in vivo experiment comprehensively indicates the therapeutic potential of Quercetagetin in RA treatment. Subsequently, to enhance validation and further explore the clinical translational potential of Quercetagetin, future studies utilizing BMMs derived from human sources are required to confirm its efficacy.

**Fig. 10:**
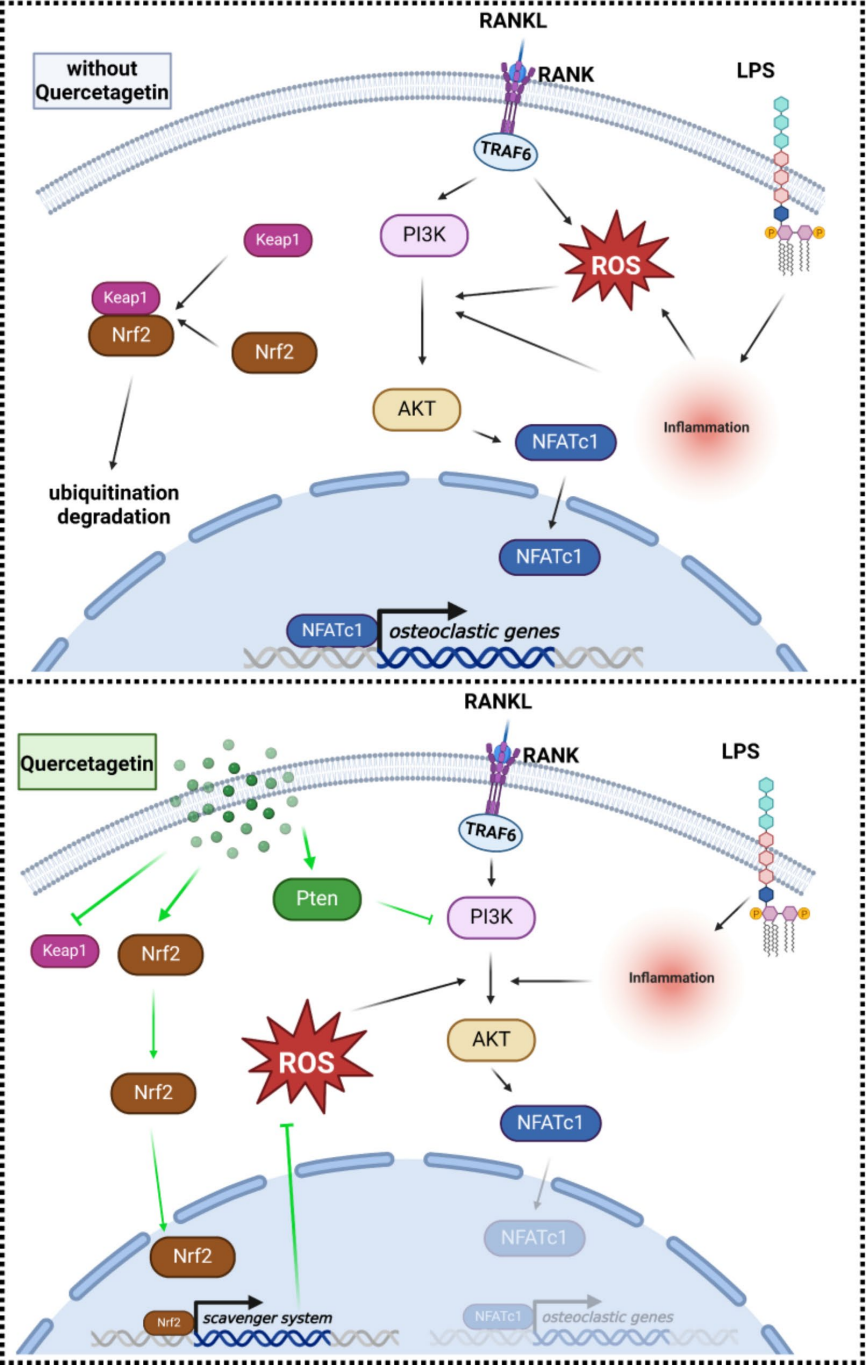
Potential working mechanism of Quercetagetin on LPS-induced osteoclastogenesis. LPS can stimulate both osteoclast differentiation and function by promoting the RANKL/RANK-ROS axis, which triggers the downstream Pten/AKT/Nfatc1 axis and subsequently activates osteoclastic gene expression. Conversely, Quercetagetin fosters Pten and Nrf2 expression, while inhibiting AKT phosphorylation and Keap1 expression, thereby attenuating LPS-induced redox imbalance and osteoclast differentiation

ROS functions as a second messenger downstream of RANKL/RANK activation, regulating subsequent signaling transductions related to osteoclast differentiation, such as the Pten/AKT/Nfatc1 axis [57–59]. Moreover, osteoclast-derived ROS overproduction under inflammatory conditions may exacerbate the local inflammatory milieu, disrupting local metabolism homeostasis and further promoting RANKL expression by fibroblasts and lymphocytes [60–62]. Concurrently, Nrf2, an essential member in the ROS scavenger system, is typically degraded through ubiquitination due to Keap1 binding. However, exposure to inducers or stressors can activate Nrf2, triggering its translocation into the nucleus and subsequently dominating the expression of antioxidant enzymes [63, 64]. Our study revealed a significant increase of the ROS level in the LPS group compared with the RANKL group. The expression of Nrf2 also displayed a mild increase, indicating a responsive activation of the antioxidant system due to excessive ROS formation due to LPS-induced inflammatory conditions as reported [65, 66]. While a previous study has highlighted the antioxidant property of Quercetagetin in relieving oxidative stress in liver injury, the specific role of which in osteoclast lineage remains to be elucidated. Our study demonstrated that Quercetagetin treatment under LPS stimulation significantly facilitated Nrf2 expression and repressed Keap1 expression, which corresponded with a significant shrinkage in ROS levels within osteoclast precursor cells. As an important signaling pathway regulating osteoclast differentiation, the Pten/AKT/Nfatc1 signaling pathway is influenced by both RANKL/RANK conjugation and ROS level, which fostering osteoclast differentiation through promoting downstream Nfatc1 expression. Our study revealed a repression of PTEN expression along with an escalation of AKT phosphorylation status under Quercetagetin treatment (Fig. 10).

Although sufficient and consistent evidence has been raised in our study to support the suppressive effect of Quercetagetin on osteoclastogenesis through regulating Nrf2/Keap1 and Pten/AKT/Nfatc1 signaling pathways, the potential involvement of other regulatory or functional factors can not be ruled out. For example, as a primary initiator and transcription factor of the antioxidant system, Nrf2 dominates the activation of multiple downstream antioxidant genes such as CAT, SOD and HO-1 [67], which may also play significant roles during LPS stimulation and Quercetagetin treatment. Concurrently, Protein disulfide isomerase (PDI), primarily situated in the endoplasmic reticulum (ER), exhibits both enzymatic and molecular chaperone activities. As per our previous research, PDIA1 and PDIA3 (ERp57) proteins from the PDI family play a central role in osteoclast differentiation through the regulation Nrf2/Keap1 signaling pathway and calcium oscillation / calcium signaling pathway [35, 36]. Consequently, the exact role of these critical regulators during osteoclastogenesis by Quercetagetin could be further investigated via gene knockdown models.

In conclusion, based on the network pharmacology analysis and biological validations, our study demonstrated that Quercetagetin could inhibit LPS-stimulated osteoclast differentiation and function through the activation of antioxidant system and inhibition of the Pten/AKT/Nfatc1 signaling pathway, therefore mitigating bone destruction in CAIA mice. Our finding provides beneficial evidence for the therapeutic application of Quercetagetin and Flos eriocauli in the prevention and treatment of RA.

## Electronic supplementary material

Below is the link to the electronic supplementary material.

Supplementary Material 1

Supplementary Material 2

Supplementary Material 3

Supplementary Material 4

Supplementary Material 5

## Data Availability

All data generated or analysed during this study are included in this published article and its supplementary information files.
